# MoTI: A Multi-Stage Algorithm for Moving Object Identification in SLAM

**DOI:** 10.3390/s23187911

**Published:** 2023-09-15

**Authors:** Changqing Hu, Manlu Liu, Su Zhang, Yu Xie, Liguo Tan

**Affiliations:** 1 School of Information Engineering, Southwest University of Science and Technology, Mianyang 621010, China; hu_changqing@mails.swust.edu.cn (C.H.); xieyu@mails.swust.edu.cn (Y.X.); 2 Robot Technology Used for Special Environment Key Laboratory of Sichuan Province, Southwest University of Science and Technology, Mianyang 621010, China; 3 School of Traffic Transportation Engineering, Central South University, Changsha 410000, China; zhangsu326@163.com; 4 Laboratory for Space Environment and Physical Sciences, Harbin Institute of Technology, Harbin 150001, China; tanliguo@mail.ru

**Keywords:** moving object detection, multi-sensor fusion, point cloud processing, SLAM algorithm

## Abstract

Simultaneous localization and mapping (SLAM) algorithms are widely applied in fields such as autonomous driving and target tracking. However, the effect of moving objects on localization and mapping remains a challenge in natural dynamic scenarios. To overcome this challenge, this paper proposes an algorithm for dynamic point cloud detection that fuses laser and visual identification data, the multi-stage moving object identification algorithm (MoTI). The MoTI algorithm consists of two stages: rough processing and precise processing. In the rough processing stage, a statistical method is employed to preliminarily detect dynamic points based on the range image error of the point cloud. In the precise processing stage, the radius search strategy is used to statistically test the nearest neighbor points. Next, visual identification information and point cloud registration results are fused using a method of statistics and information weighting to construct a probability model for identifying whether a point cloud cluster originates from a moving object. The algorithm is integrated into the front-end of the LOAM system, which significantly improves the localization accuracy. The MoTI algorithm is evaluated on an actual indoor dynamic environment and several KITTI datasets, and the results demonstrate its ability to accurately detect dynamic targets in the background and improve the localization accuracy of the robot.

## 1. Introduction

SLAM is the basis for robots to engage in exploratory work in unknown environments, and it is also an essential manifestation of robot autonomy. In standard SLAM algorithms, the assumption of a static environment is very typical, while in more cases, the robot is exposed to a dynamic environment. Moving objects in the background will act as noise, which may degrade the sensors and inhibit the system’s positional estimation robustness. Furthermore, the environment perception capability is a facilitator for later applications in service robots or autonomous driving. Therefore, detecting and eliminating dynamic obstacles is vital for improving the effectiveness of localization and map construction.

At present, conventional methods for target detection include vision-based [1], laser-based [2], and vision-laser fusion-based methods [3]. There are only a small number of published research studies concerning lidar SLAM systems dealing with moving objects, which are based on a post-processing approach to project point clouds into range images and use the current frame of the point cloud for matching with submap to identify outliers. However, these methods are still limited by the environment during real-time processing. A more general system is to combine laser data or image data with a neural network to recognize and label dynamic objects using semantic segmentation [4,5]. Compared with other target detection methods, the supervisor learning method YOLOv5 can effectively improve the efficiency and accuracy of visual identification in dynamic environments [6].

Single-sensor systems may result in degraded map quality due to limited sensing capabilities. In contrast, multi-sensor information fusion acquires different feature information from different sensors, which enriches the measurement information and is helpful for environment perception and state estimation. However, there are still several challenges that have to be overcome in the system design process. On the one hand, since laser point cloud data are sparse and image data are a complete description of a certain angle range, the difference in information sources leads to more difficulty in data fusion. On the other hand, a proper judgment mechanism has to be introduced to prevent point clouds from moving objects from being misjudged as static points.

Therefore, a suitable multi-sensor fusion system combined with supervisor learning for point cloud processing can be designed to effectively detect dynamic features. In this paper, an algorithm for dynamic point cloud detection that fuses laser and visual data is proposed. By fusing the detection information of YOLOv5 and point cloud data, we design a judgment mechanism for moving point clouds based on statistical information through multiple stages of point cloud processing. In this manner, we achieve the detection and separation of dynamic point clouds. Static point clouds are used as input for SLAM to improve the accuracy of localization and point cloud alignment. The main contributions of this study are as follows: (1) A point cloud processing method was proposed based on two-stage processing to achieve the detection and marking of point clouds on moving objects. (2) We apply the MoTI algorithm to the front end of LOAM to achieve a complete marking of the point cloud on moving objects. Based on this, we improve the localization accuracy and mapping effect of the system. (3) Simulation and indoor dynamic scenario experiments were carried out, respectively, and the results show good performance in identifying moving targets and robot localization.

The paper is organized as follows: Section 2 discusses the related research work on SLAM in dynamic scenarios. Section 3 provides a detailed description of the theory and relevant details of the MoTI algorithm. The results of a series of experimental tests and evaluations of the system are presented in Section 4. Finally, the conclusions are drawn in Section 5.

## 2. Related Work

SLAM algorithms for dynamic scenes are mainly divided into vision-based and laser-based processing approaches. The current mainstream methods mostly use vision or laser information combined with deep learning methods to supply semantic information to the environment. The literature on fusing laser and visual identification information in SLAM for moving target detection is limited.

### 2.1. Vision-Based Approaches

Some SLAM algorithms based on static assumptions of the environment are also robust in dynamic environments, such as ORB-SLAM [7], which generally uses a random sample consensus (RANSAC) algorithm to reject anomalous feature associations. However, dynamic features will inevitably be involved in the pose calculation of the robot. Typical vision-based processing methods include geometric, optical flow, and deep learning. Litomisky et al. [8] adopted the technique of multi-view geometry to judge whether the object is moving according to the relative position changes of different objects in two views. Sun et al. [9,10] used vector quantized depth images to segment moving objects based on RGBD-SLAM.

The methods of deep-learning can effectively identify common dynamic targets in the environment. Fan and Zhang et al. proposed Blitz-SLAM [11], which combines semantic information and geometric information of masks and depth maps. The semantic information is obtained by BlitzNet, which acts on the front end of ORB-SLAM, similarly including literatures [12,13]. Xiao et al. proposed Dynamic-SLAM [14], which is based on the SSD method to solve the problem of dynamic features missing detection compensation, judge dynamic feature points according to the principle of dynamic target feature mismatch under violation of geometric constraints, and propose a selective tracking algorithm. Liu et al. proposed RDS-SLAM [15], adding a semantic thread and an optimization thread based on ORB-SLAM3 for robust tracking and mapping in real-time dynamic environments.

In [1], RGB images are used as input data, and scene flow and semantic segmentation are combined. Dynamic and non-dynamic objects are judged according to the threshold of scene flow intensity, which can realize the localization, mapping, and tracking of highly active objects on the road [16]. In [17], the same method of optical sampling flow is used to estimate the three-dimensional motion field, combining a rigidity-transform network (RTN) with a two-dimensional optical flow network. The dense scene flow is subtly converted into a least squares problem. For the complex dynamic environment, the solution of how to accurately and effectively detect the position and range of moving objects to avoid the influence of moving objects on SLAM is not complete.

### 2.2. Laser-Based Approaches

Compared with vision-based methods, laser data can describe the geometric information of space more accurately. Laser-based moving object processing includes dynamic target detection [18] and tracking, and dynamic target detection and filtering [19]. This article focuses on the latter. Chen et al. [19] exploit sequential range images from a rotating 3D LiDAR sensor as an intermediate representation combined with a convolutional neural network and runs faster than the frame rate of the sensor. Demim et al. [20] proposed ASVSFSLAM based on SVSF [21], adding covariance to optimize the filter, and using a heuristic strategy to select the features to be removed. Similarly, ref. [22] constructs an occupancy map as a filter to remove dynamic points from the laser point cloud, and combines the target detection method AVOD (aggregate view object detection) to classify the identified object. The voxel traversal algorithm is adopted in this literature to accelerate the map update. To perceive changes in the environment, Einhorn et al. [23] proposed lifelong SLAM based on a standard distribution transform map (NDT map), which uses attitude map optimization to enhance localization and maintain the real-time performance of the system by constructing attitude maps. This method can detect and track moving objects, while updating the map online and judging whether it is a dynamic area according to the grid occupancy probability and the length of the grid occupancy period.

Lim et al. [2] proposed the ERASOR method based on the fundamental assumption that moving objects are connected to the ground, and proposed pseudo-occupancy to represent the degree of occupancy in cell space while combining ground plane fitting and checking the difference of transformed points to distinguish dynamic points from static ones. Pfreundschuh et al. [24] proposed an end-to-end network (novel end-to-end occupancy grid-based pipeline), which can automatically mark dynamic objects in point clouds and effectively improve the performance of odometry. Both of these methods are based on grid probability for dynamic object detection. To enhance the ability of environmental perception, Liang et al. [25] integrated 2D, 3D, and bird’s eye view (BEV) with three different levels of information to obtain better feature representation for moving target detection, and achieved pixel-level matching between laser point clouds and BEV feature maps based on the geometric features of the distance between different levels. Although laser data can accurately describe the geometric information of the environment, it is still difficult to detect moving objects in the environment with point cloud alone.

## 3. Approach for Localization and Mapping Based on Moving Object Detection

### 3.1. Overview of the Proposed System

**Figure 1:**
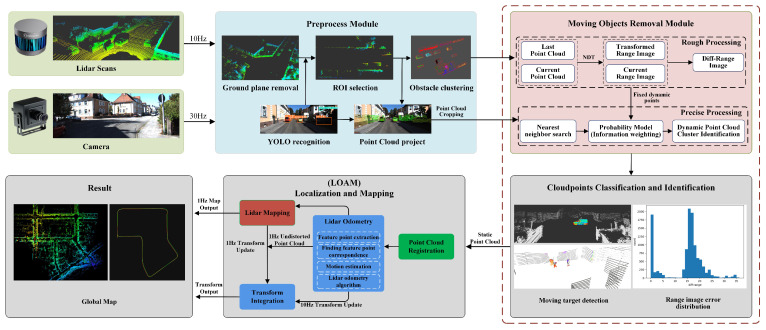
Software System Overview. The MoTI algorithm is used for moving object removal on the basis of the pre-processing module. In the moving object removal module, the detection of dynamic point clouds is completed in two stages, rough processing and precise processing, during which a probabilistic model is constructed by means of statistics and information weighting fusion.

The existence of moving objects in the environment causes the point cloud matching process to unavoidably substitute dynamic points into the iterative computation, which leads to a bias in the state estimation. Therefore, this paper proposes the MoTI algorithm that removes dynamic objects based on the fusion of lidar and visual identification information during two stages, which is applied to point cloud processing in low-dynamic scenarios. In order to improve the computational efficiency, we complete the operations of ground point cloud rejection, region of interest selection, point cloud clustering, and image detection data encapsulation before the rough processing stage. The moving object removal module is a major part of the MoTI algorithm, combining statistical ideas and information weighting methods to construct probabilistic models [26] in both the coarse and fine processing stages. Subsequently, we apply the MoTI algorithm to the front-end of the LOAM algorithm to improve the localization accuracy and mapping effect of the system. The detailed processing flow of the data is shown in Figure 1.

### 3.2. Preprocess Module

The main function of this module is to process laser point cloud and image data including lidar–camera extrinsic calibration, ground plane removal of point cloud, region of interest (ROI) selection, obstacle clustering, and YOLO real-time target detection based on images and point cloud projects.

#### 3.2.1. Lidar–Camera Extrinsic Calibration

**Figure 2:**
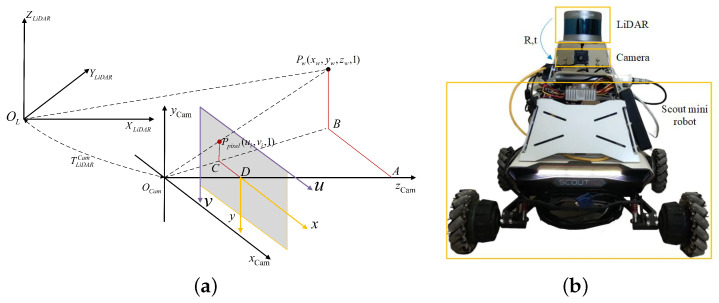
Transformation of the coordinate system. (**a**) Transformation of point cloud to pixel coordinate. (**b**) Multi-sensor system and mobile robot platform.

Before projecting the point cloud data onto the image frame at the corresponding moment to fuse the camera and laser information, it is necessary to calibrate the lidar–camera extrinsic parameters. The conversion of the point cloud to the pixel coordinate system is shown in Figure 2a. We define *s* as the scaling factor, K as the internal camera matrix, $R_{rect}$ as the camera distortion correction matrix, and set $T_{l}^{c}$ as the transformation matrix from lidar to camera. Since transforming 3D points to 2D images drops the Z-axis information and leads to nonlinearity, we set $p_{i}={\left(x_{i},y_{i},z_{i},1\right)}^{T}$ as the point coordinates. The pixel position $\left(u_{i},v_{i},1\right)$ is obtained according to the following equation:

(1) $$s{\left[\begin{array}{ccc} u_{i} & v_{i} & 1 \end{array}\right]}^{T}=K*R_{rect}*T_{l}^{c}*p_{i}$$

(2) $$K=\left[\begin{array}{ccc} f_{x} & 0 & u{}_{c}\\ 0 & f_{y} & v_{c}\\ 0 & 0 & 1 \end{array}\right],T_{l}^{c}={\left[\begin{array}{cc} R_{l}^{c} & t_{3\times 1}\\ 0_{1\times 3} & 1 \end{array}\right]}_{4\times 4}$$

where $t_{3\times 1}$ is the translation from Lidar to camera and the matrix $T_{l}^{c}$ is a crucial parameter. This paper follows the approach in [27] to constrain the relative positions and orientations of the lidar and camera through the normal of the checkerboard plane and the 3D points located on the surface of the checkerboard. These constraints can be used to construct a nonlinear optimization problem that is eventually solved to obtain the external calibration parameters. Then, the SCOUT-mini robot is selected as the mobile robot verification platform, as shown in Figure 2b.

#### 3.2.2. Ground Plane Removal

A large number of ground point clouds will greatly increase the computational cost of state estimation. To remove the ground better, the RANSAC algorithm [28] combined with the region-growing algorithm is used to fit the ground. Firstly, the point cloud is sorted according to the Z-axis coordinates from most minor to most significant, the average value of the Z-axis coordinates $Z_{mean}$ of the first n points, and $\left(\bar{x},\bar{y},\bar{z}\right)$ is used as the seed point. A reasonable threshold $\left\{\left.Z_{i}\leq Z_{mean}+Z_{threshold\_seed}\right|i\leq Cloudsize\right\}$ is set to extract the seed region from the point cloud. The ground point cloud can be initially obtained by iterative search based on whether the Z-axis coordinates of the point cloud meet the threshold interval. To solve the plane equation of the ground, we substitute $\left(\bar{x},\bar{y},\bar{z}\right)$ into ax+by+cz+d=0; that is, find the normal plane vector $\vec{n}=\left(a,b,c\right)$ and constant value *d*. The SVD decomposition of n points can obtain $\vec{n}$. We set the distance threshold threshold_dist, and further separate the non-ground points according to whether the point cloud conforms to the plane equation. The non-ground points are satisfied for the following condition.

(3) $$\left\{{\vec{n}}^{T}*p_{i}+d>threshold\_dist\right\},\left\{\left.p_{i}={\left(x_{i},y_{i},z_{i}\right)}^{T}\right|i\leq Cloudsize\right\}$$

Extensive experimental results indicate that when threshold_dist=0.12, $Z_{threshold\_seed}$ = 10, a more complete ground point cloud segmentation can be achieved.

#### 3.2.3. Region of Interest Selection

This paper combines the visual information to improve the consistency between the laser data and the visual observation information to crop the point cloud. For this reason, we only select the points that can be projected into the image. After performing the lidar–camera coordinate transformation according to Equation (1), the point cloud can be projected into the pixel coordinate system. The laser points that satisfy the threshold interval $u_{i}\in \left[0,width\right]$, $v_{i}\in \left[0,height\right]$ are selected as the points of interest. Based on the external calibration results, the horizontal field of view (fov) of the LiDAR after projecting the point cloud onto the image is $\left[-39.5^{\circ },39.5^{\circ }\right]$, and the vertical fov is $\left[-24.8^{\circ },2.0^{\circ }\right]$.

#### 3.2.4. YOLOv5 and Obstacle Clustering

As an efficient target detection model, YOLOv5 is equipped with the ability to perform real-time target detection on video stream data [29], and can accurately identify 80 common objects. In this study, only the typical subjects are detected such as person, bicycle, car, truck etc.

**Figure 3:**
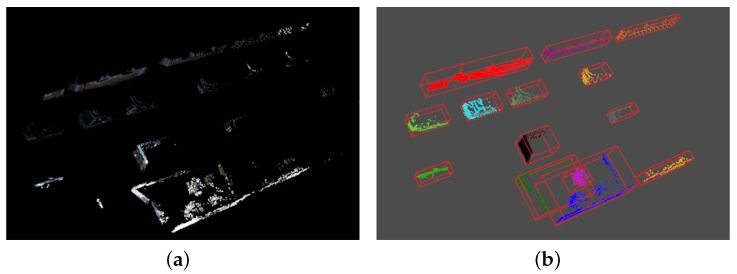
The result of the point cloud with Euclidean clustering. (**a**) The original point cloud. (**b**) The point cloud clustering effect.

After the above processes, the moving objects will be independent as point cloud clusters, so we use Euclidean clustering to cluster the point clouds based on the Euclidean distance between points, and cluster each frame into multiple groups to identify the moving obstacles better. We select a seed point $p_{i}$ and use KD-Tree to search for the *n* closest points with distance calculated as follows:

(4) $$d\left(p_{a},p_{b}\right)=\sqrt{{\left(x_{a}-x_{b}\right)}^{2}+{\left(y_{a}-y_{b}\right)}^{2}+{\left(z_{a}-z_{b}\right)}^{2}}$$

(5) $$p_{j}\in \left\{\begin{array}{c} M\\ others \end{array}\begin{array}{c} \;d(p_{i},p_{j})<r\\ \;d(p_{i},p_{j})>r \end{array}\right.,j=1,2,\cdots ,n.$$

where *r* is a known distance threshold. Next, we select a point $p_{j}$ from *M* and repeat the previous steps until no point is added to the set to end the search. The point cloud clustering effect is shown in Figure 3.

#### 3.2.5. Transformation of Point Cloud to Range Image

**Figure 4:**
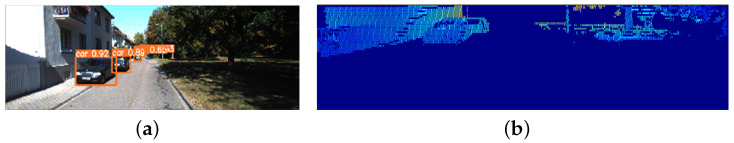
The effect of point cloud to range image after ground removal. (**a**) Image data from the KITTI dataset. (**b**) Result of converting the point cloud at the same moment as (**a**) into a range image after ground removal. Closer points are blue, and the further ones are yellow.

In the literature [18], the point cloud frame is converted into a range image, which is a way to sparse the points information. The image represents the elevation and offset angle of the laser point cloud in the horizontal and vertical planes. Based on the above, the ground is removed in this paper, and the point clouds cropped for the region of interest will be clustered. Therefore, converting the point cloud to a range image will be more effective in visualizing the depth variation of the point cloud. According to the equation $depth=\sqrt{\left(x^{2}+y^{2}+z^{2}\right)}$, the depth values are stored in each pixel of the range image, while ground points and points at an infinite distance are valued at zero. The result after transformation is shown in Figure 4.

### 3.3. Moving Objects Removal Module

#### 3.3.1. Rough Processing for Moving Objects Removal

Rough processing stage is the first stage of the MoTI algorithm. The purpose of this stage is to quickly classify the point cloud roughly into static and dynamic points in order to improve the speed and accuracy of the nearest neighbor search in the second stage of precision processing. Therefore, we convert the point cloud into a range image and store it as a matrix for easy mathematical operations.

Unlike the ray projection method and grid occupancy method [23,30], this paper adopts the viewpoint visibility method to detect the dynamic point cloud and downscale the 3D laser data into 2D image data. We determine dynamic points by performing matrix subtraction of the range image. By matching the point clouds of adjacent time steps, we obtain the approximate dynamic range based on the distribution of pixel depth variations. The depth of points changes according to the object’s different movement states.

**Figure 5:**
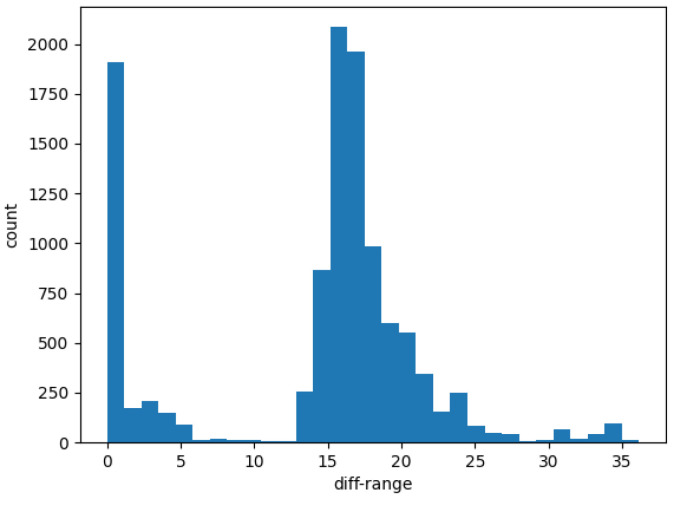
The result of range image error distribution (the zero value of the horizontal coordinate means the ground point).

Since the vehicle is also moving, we must align and match two adjacent points of the point cloud to determine whether the depth of the same point in space changes at adjacent moments. The point clouds of adjacent moments are iteratively aligned by normal distributions transformation (NDT) [31]. NDT uses the probability density function (PDF) of each voxel for probability matching, and the point x is obtained by distribution sampling. The likelihood of the point x is assumed to be

(6) $$\rho \left(x\right)=\frac{1}{{\left(2\pi \right)}^{D/2}\sqrt{\left\lfloor \Omega \right\rfloor }}\exp\left(-\frac{1}{2}{\left(x-\mu \right)}^{T}\Omega^{-1}\left(x-\mu \right)\right)$$

(7) $$\mu =\frac{1}{n}\sum_{i=1}^{n}p_{i},\Omega =\frac{1}{n-1}\sum_{i=1}^{n}\left(p_{i}-\mu \right){\left(p_{i}-\mu \right)}^{T}$$

where μ and Ω are the mean vector and covariance matrix of the points within the voxel. The aim is to maximize the likelihood that the points of the current frame after the positional transformation *T* lie on the surface of the reference frame,

(8) $$T^{*}=\underset{T}{\mathrm{argmax}\psi }\;=\underset{T}{\mathrm{argmax}}\;\underset{k=1}{\prod^{n}}\;\rho \left(Tp_{i}\right)=\underset{T}{\mathrm{argmin}}\;-\sum_{k=1}^{n}\log\rho (Tp_{i})$$

(9) $$\bar{\rho }\left(x\right)=c_{1}\exp\left[-\frac{1}{2}{\left(x-\mu \right)}^{T}\Omega^{-1}\left(x-\mu \right)\right]+c_{2}\xi$$

where $c_{1}$ and $c_{2}$, which can enable $\bar{\rho }\left(x\right)$ to integrate to 1 in voxel space, are constants, while ξ is the scale factor of the outliers. We can make the point clouds of adjacent frames approximately coincide and obtain the transformation matrix $T_{k-1}^{k}$ of adjacent moments.

(10) $$T_{k-1}^{k}=T_{k-1}^{-1}*T_{k},\Delta T=T_{k-1}^{k}$$

where $T_{k-1}$, $T_{k}$ are the state matrices of k−1 and *k* moments, respectively. The following conditions must be satisfied for the same static points in adjacent frames.

(11) $$p_{j}^{k-1}-{\left(p_{i}^{k}\right)}^{-1}=p_{j}^{k-1}-\Delta T*p_{i}^{k}<\varsigma$$

where ς is a minimal quantity. In contrast, the fluctuation of the depth value of the dynamic point will deviate from the threshold value ς. According to the results of the diff-range image error distribution in Figure 5, we assume that the measurement noise obeys a Gaussian distribution ${}^{Static}I_{diff}^{k}∼N\left(m,\sigma^{2}\right)$, the elements of the diff-range matrix are sorted from small to large, and the top n depth values are taken to calculate the mean *m*, as well as the variance $\sigma^{2}$. In order to ensure better matching accuracy, the point cloud at the current moment is converted to the last moment under the coordinate system, and the range matrix is made to differ later.

**Figure 6:**
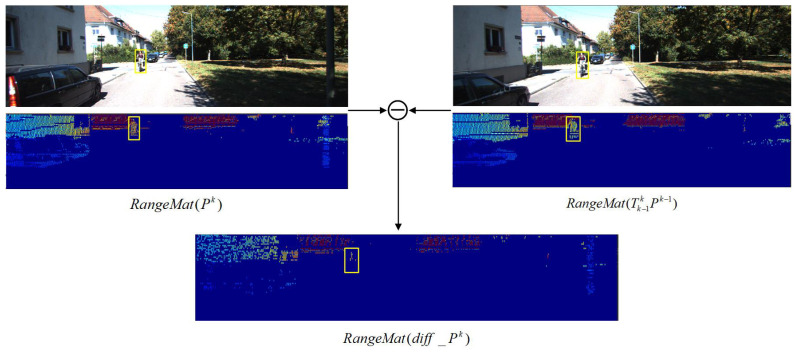
Dynamic point removal process based on the error of range image.

We set the current frame point cloud as $P^{k}$ and the previous frame point cloud as $P^{k-1}$. Then, the previous frame point cloud converted to the current frame coordinate system as $T_{k-1}^{k}P^{k-1}$. The function of the range image matrix error is as follows, and the result is shown in Figure 6.

(12) $$RangeMat\left(P^{k}\right)-RangeMat\left(T_{k-1}^{k}P^{k-1}\right)=RangeMat\left(\mathrm{diff}\_P^{k}\right)$$

where $\mathrm{diff}\_P^{k}$ is the error matrix of the point cloud depth after making the difference, and RangeMat(•) converts the point cloud to a 64×900 depth matrix.

$RangeMat(\mathrm{diff}\_P^{k})$ contain both dynamic and static points. By judging whether the matrix elements satisfy the above Gaussian distribution, the dynamic and static points can be initially classified.

(13) $$P^{k}=\left\{{}^{Static}P^{k}⋃{}^{Dynamic}P^{k}\right\}$$

Since the way is a statistical result, there is a statistical error, and some fixed points exist in the filtered dynamic points, so further selection is needed.

#### 3.3.2. Precise Processing for Moving Object Removal

The precise processing stage is the second stage of the MoTI algorithm. The objective of this stage is to search the rough processed point cloud for the nearest neighbor points and establish the movement evaluation probability model based on the search results and visual identification for each obstacle cluster. As the point cloud is filtered during the rough processing stage, the data dimension is further reduced, and the storage of the point cloud index is performed using map value pairs to enhance data processing efficiency. This ensures that the two frames of the point cloud after rough processing can avoid the data explosion problem after downsampling.

**Figure 7:**
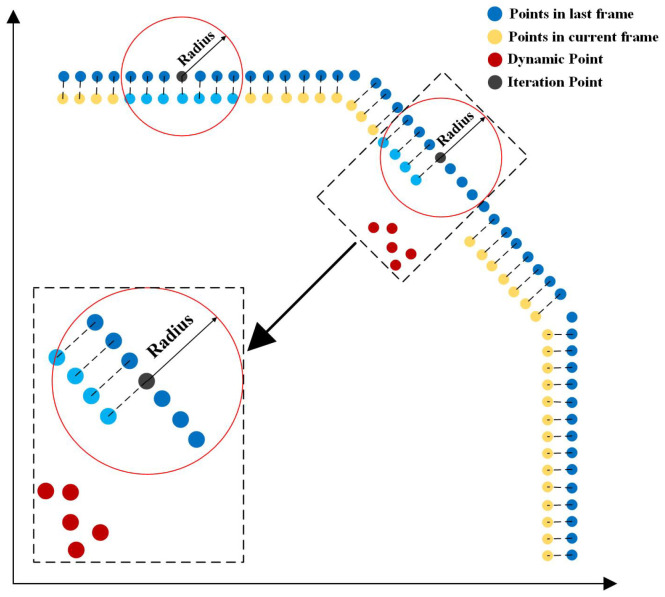
The filtering strategy for dynamic points.

To achieve this, we use K-nearest neighbor search and radius search, as shown in Figure 7. For the iterative points of static objects in the previous frame, we search for the laser points corresponding to the current frame within their radius range after matching. We record the point cloud indexes in the radius range and the number of times searched.

The nearest neighbor search is performed on the point cloud sets ${}^{Static}P^{k}$ and ${}^{Dynamic}P^{k}$ after the first stage of processing. For the point cloud frame $P^{k}=\left\{\left.p_{0}^{k},p_{1}^{k},\ldots ,p_{i}^{k}\right|i<N-1\right\}$ at moment *k*, we iterate $p_{i}^{k}$ for the radius search. The points out of the radius are considered dynamic points, with statistical results stored in the form of a map with the structure of (m,count), where *m* is the serial number of the point cloud cluster after clustering, and count is the number of dynamic points counted in the corresponding cluster. We introduce a Bernoulli-distributed random variable $\xi_{i}^{k}$ to determine whether the point $p_{i}^{k}$ falls within the YOLO recognition boxes.

(14) $$P\left(\xi_{i}^{k}=1\right)=q_{i}^{k},P\left(\xi_{i}^{k}=0\right)=1-q_{i}^{k},0\leq q_{i}^{k}\leq 1$$

For the accurate identification of dynamic points, we define the dynamic probability of the clustered point cloud as follows:

(15) $$pro_{j}^{k}=f\left(p_{visual-identify}+p_{k-search}\right)$$

(16) $$p_{visual-identify}=\sum_{i=0}^{N-1}\omega_{i}/count,p_{k-search}=count/S_{j},\omega_{i}=P\left(\xi_{i}^{k}=1\right)$$

(17) $$\mathrm{point}\;\mathrm{cloud}\;\mathrm{cluster}\;j=\left\{\begin{array}{c} static\;\;if\;pro_{j}^{k}\leq \tau \\ dynamic\;otherwise \end{array}\right.$$

where the f(⋅) is a nonlinear weighting function, the variable τ is introduced as a critical value to determine whether the cluster belongs to dynamic objects, and $S_{j}$ is the total number of points in the jth cluster. Then, the clustered point clouds can be judged according to Formula (15). Further, the separated point clouds are used as the input of the SLAM algorithm to construct a global map.

### 3.4. Localization and Mapping

This module consists of four parts: point cloud registration, lidar odometry, lidar mapping, and transform integration. The motion state drift error and point cloud alignment problem are effectively solved after the previous stages of data processing. The processing of the moving objects improves the accuracy of the point cloud alignment, which effectively reduces the motion state drift error and the cost of numerical calculation, ultimately achieving more accurate localization. The algorithm framework of LOAM [32] mainly includes feature point extraction, finding feature point correspondence, motion estimation, lidar odometry algorithm, and lidar mapping algorithm.

#### 3.4.1. Feature Point Extraction

The feature points are extracted based on curvature, and the current frame point cloud is divided into edge points and plane points. Due to the removal of ground points, the number of planar features is reduced heavily. The curvature is calculated as follows:

(18) $$cur=\frac{1}{\left|S\right|∥P_{i}^{k}∥}∥\sum_{j\in S,j\neq i}\left(P_{i}^{k}-P_{j}^{k}\right)∥$$

where *S* is the set of continuous points returned from the current frame point cloud. To ensure the quality of feature points, in the feature extraction process, the feature points in the three cases of weak laser point intensity, the presence of occlusion near the scanned point, and too close to the laser will be ignored. The final obtained points are classified into the edge point set and the planar point set, respectively.

#### 3.4.2. Finding Feature Point Correspondence

The evaluation of the degree of data association is described based on the point-to-line distance and the point-to-plane distance. Assuming that there are three planar feature points *i* in the current frame, and *j*, *l*, and *m* in the two adjacent line bundles in the previous frame, which constitute point-to-plane distance constraints, and are characterized as follows.

(19) $$d_{plane}=\frac{\left|\begin{array}{c} \left(P_{i}^{k+1}-P_{j}^{k}\right)\\ \left(P_{j}^{k}-P_{l}^{k}\right)\times \left(P_{j}^{k}-P_{m}^{k}\right) \end{array}\right|}{\left|\left(P_{j}^{k}-P_{l}^{k}\right)\times \left(P_{j}^{k}-P_{m}^{k}\right)\right|}$$

Similarly, assuming that there is a current frame edge point $P_{i}^{k+1}$, the nearest neighbor point $P_{j}^{k}$ in the previous frame and an edge point $P_{l}^{k}$ on the line bundle adjacent to point $P_{j}^{k}$. These three constitute a point-line distance constraint, which is characterized as follows:

(20) $$d_{edge}=\frac{\left|\left(P_{i}^{k+1}-P_{j}^{k}\right)\times \left(P_{i}^{k+1}-P_{l}^{k}\right)\right|}{\left|P_{j}^{k}-P_{l}^{k}\right|}$$

#### 3.4.3. Motion Estimation

During the laser scanning process, each laser point may generate motion drift as the robotic platform is constantly moving. To solve this problem, the movement compensation of laser points is proposed, which is expressed as follows:

(21) $$T_{k+1}^{i}=\frac{t_{i}-t_{k+1}}{t-t_{k+1}}T_{k+1}$$

where $t_{i}$ is the timestamp of the point of the current frame of the point cloud, and $T_{k+1}^{i}$ is the pose transform between $\left[t_{k+1},t_{i}\right]$. This linear interpolation can effectively solve the motion distortion of laser points.

Then, the nonlinear optimization function is constructed. Based on the point–line distance as well as the point–plane distance constraints, the following equations can be constructed:

(22) $$f_{edge}\left(P_{i}^{k+1},T_{k+1}\right)=d_{edge},i\in edge\;point\;set$$

(23) $$f_{plane}\left(P_{i}^{k+1},T_{k+1}\right)=d_{plane},i\in planar\;point\;set$$

By combining the above two equations, the final optimization equation $f(T_{k+1})=d$ can be obtained, and the optimal solution can be calculated by using Levenberg–Marquardt (LM) optimization.

#### 3.4.4. Lidar Odometry and Mapping Algorithm

The odometry estimation is computed iteratively by adjacent-frame scan-to-scan matching. The efficiency and speed of point cloud alignment will be greatly improved after moving targets and a large number of ground points are removed. The odometry estimation is updated by two ICP (iterative closest point) alignments, which calculate the positional transformation and perform nonlinear optimization for edge points and planar points, respectively.

In contrast, the mapping frequency is slow, and the map alignment is performed at 1 Hz. The mapping algorithm uses scan-to-map alignment to match the current frame point cloud with the submap, similar to Equations (19)–(23). The feature constraints are constructed for nonlinear optimal solution. Moreover, since the point cloud clustering is at the front end, the obstacles that are independent in the final space are separated completely, and then obstacles on the output map will be easily identified.

Therefore, after the previous multiple stages of point cloud processing and downsampling work, scan-to-scan and scan-to-map matching speed will be significantly improved, and after the moving targets in the point cloud are separated, the alignment accuracy is further improved.

## 4. Experiments

To evaluate the performance of the MoTI algorithm, we conducted dynamic point cloud detection and recognition, as well as map construction based on the KITTI dataset (containing point cloud, visual data, and external calibration information) and actual scenario. We also compared the localization performance with LOAM and LeGO-LOAM algorithms and performed a comparative analysis of the localization effect of the algorithm using the EVO tool. It should be noted that the LOAM algorithm cannot detect dynamic point clouds, and therefore, leaves noise blocks on the map during the mapping process; LeGO-LOAM is able to filter the point clouds on moving objects by extracting specific feature points from the segmented point cloud.

The overall experiment consists of three parts. The first part illustrates the effectiveness of the two stages of the MOTI algorithm for dynamic point cloud processing with the KITTI dataset. The second part is an experiment on detecting dynamic points in real scenario. The third part is the experiment of localization and mapping based on the completed detection of moving objects.

### 4.1. Dynamic Point Removal and Moving Objects Detection

**Figure 8:**
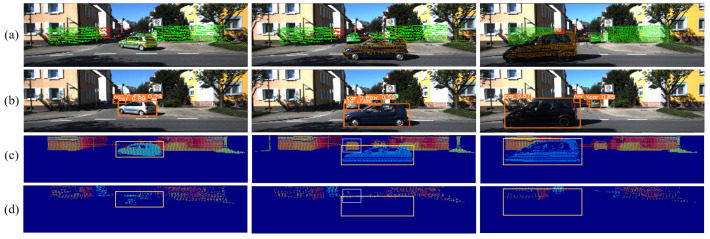
The result of rough process with moving object detection and rejection. (**a**) The effect of point cloud and image fusion. (**b**) The result of YOLO detection. (**c**) Range image of the point cloud at the same moment as (**a**,**b**). (**d**) The final dynamic rejection result of moving object.

Figure 8 displays the dynamic rejection result based on range image processing at three different moments in the neighborhood during rough processing. The analysis, combined with Figure 6, demonstrates that the MoTI algorithm can recognize moving objects of different scale sizes and reject corresponding point clouds. However, some fixed points are misidentified, which highlights the need for precise processing in the next stage.

**Figure 9:**
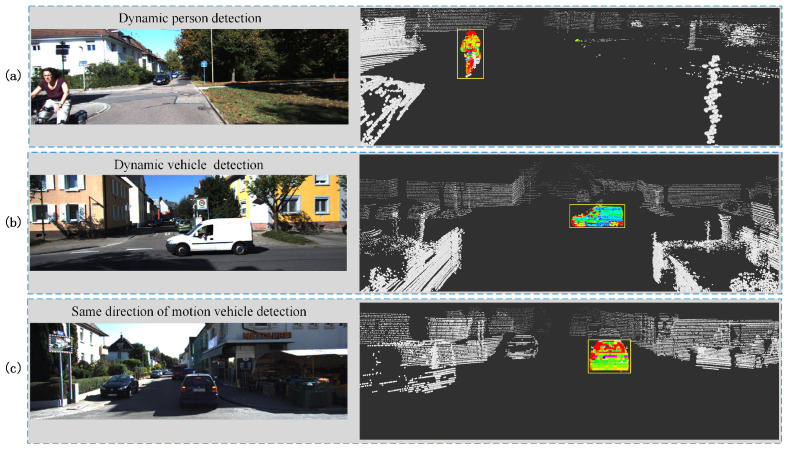
The result of precise process with moving object detection. (**a**) On-coming pedestrian detection results during moving. (**b**) Detection of lateral moving vehicles while waiting for trains at intersections. (**c**) Detection of vehicles moving in the same direction.

Figure 9 shows the results of detecting different moving objects during precise processing. The number of false positives in the static point cloud is significantly reduced, and the overall dynamic detection effect is greatly improved after the precise processing. As shown in Figure 9, moving vehicle and person can be accurately detected.

### 4.2. Real Scenario Experiments of Moving Objects Detection

**Figure 10:**
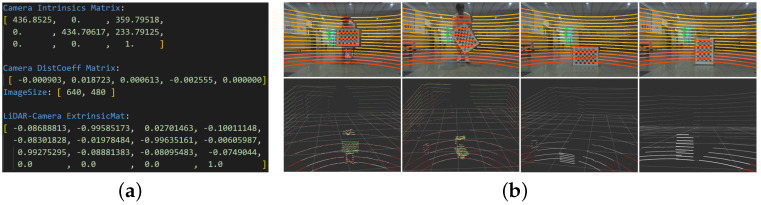
Results of the joint external reference calibration. (**a**) The calibrated internal and external parameters of the lidar–camera system on the scout-mini robot. (**b**) Lidar–camera calibration performance results. The checkerboard size is 7*10, and the side length of a single grid is 50 mm.

The calibration of the sensor external parameters, and the result are shown in Figure 10a. The experimental platform is the SCOUT-mini robot with RS-LiDAR-16 LiDAR and a USB monocular camera.

**Figure 11:**
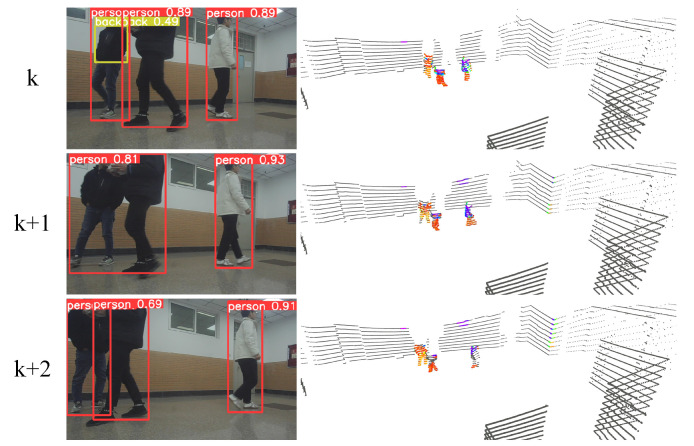
Detection of moving objects by mobile robots with continuous frames in real scenarios.

Figure 11 shows three adjacent moments of observations from top to bottom. The YOLO recognition results are highly consistent with the dynamic recognition of the point cloud, and there is continuity in time and space. Dynamic objects in the area detected by YOLOv5 are fully identified, and their corresponding moving targets in the point cloud are accurately detected.

Table 1 shows the dynamic point cloud detection results of three consecutive frames in the actual scenario in Figure 11, where “Dynamics number” is the frequency of point clouds identified by the MoTI algorithm, “Truth number” is the frequency of point clouds of ground obstacles output after clustering, and “Identification rate” is the ratio of the two, which indicates the identification accuracy of dynamic points. As shown in the table, the average identification accuracy of moving objects in natural low dynamic scenes is about 93%, proving the MOTI algorithm’s effectiveness.

Combining two experimental results from the KITTI dataset and the low dynamic dataset in natural scenes, the performance of the MoTI algorithm for detecting point clouds on moving objects is demonstrated.

To better compare the performance of the algorithm, we used the KITTI dataset with ground truth for verification of the performance of the localization algorithm. Meanwhile, we conducted similar tests in real scenarios and analyzed the performance of the MoTI algorithm for localization. Hence, we conducted the following experiments.

### 4.3. Experiments of Localization and Mapping

Outlier points have been accurately detected based on previous processes. The filtered point cloud is used as input for LOAM to improve localization accuracy and mapping effects. This study was conducted on the Lenovo ThinkPad T490 hardware platform, equipped with an Intel^®^ Core^TM^ i7-8565U CPU and NVIDIA GeForce MX250 for testing and experiments.

Table 2 shows the absolute trajectory error (ATE) of LOAM, LeGO-LOAM (without loopclosure detection module), and the proposed algorithm for KITTI datasets 00, 06, and 10, respectively. In all three datasets, there are different numbers of moving objects. It should be noted that the ATE includes both translation and rotation errors. The EVO tool is used to calculate the statistical error between the trajectory and the ground truth, including the mean value, median value, minimum value, root mean square error (RMSE), and standard deviation (STD). When using RMSE as the evaluation standard for localization accuracy, the results demonstrate that the proposed algorithm has better accuracy than the LOAM and LeGO-LOAM. The proposed algorithm improves the localization accuracy by about 12.8% on average over the three data sets compared to the LOAM without a moving target detection module. In Table 3, which shows the relative pose error (RPE) of the three algorithms in different data sets, using RMSE as the basis for comparison. The analysis of the results in the table shows that the local localization accuracy of the proposed algorithm in this paper is significantly improved compared to LOAM and LeGO-LOAM.

**Figure 12:**
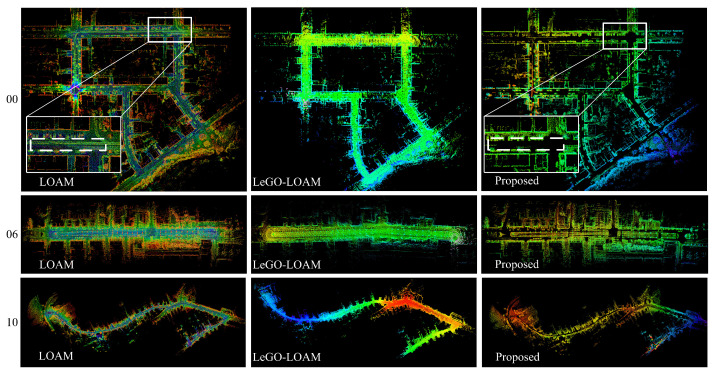
Comparison of localization and mapping result. The “00”, “06”, and “10” are the dataset sequences of KITTI.

Figure 12 shows the map construction results of the three algorithms in the datasets 00, 06, and 10. The point cloud maps of the 00 sequence show that the proposed algorithm is optimized to a certain extent for movement ghosting in the maps. Compared to the LOAM and the LeGO-LOAM, the map constructed by the proposed algorithm is relatively sparse, but the separation of obstacles on the ground is complete.

**Figure 13:**
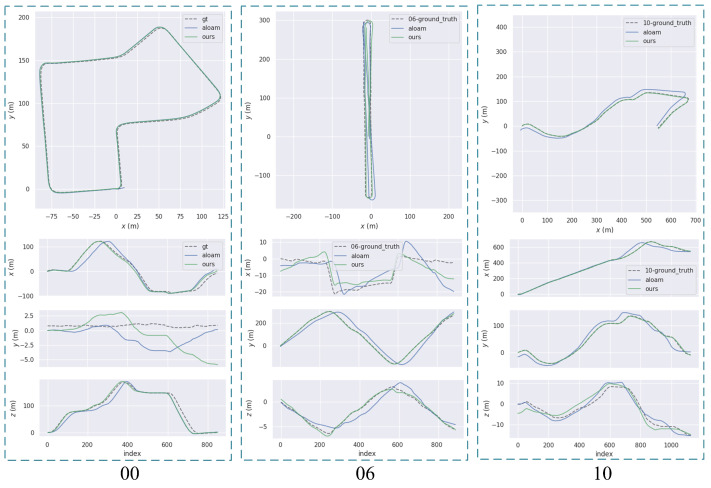
The error between trajectory and ground truth.

Figure 13 shows the trajectory poses of the algorithm odometry, and the proposed algorithm is compared with the LOAM localization data. The analysis in the figure shows that the trajectory of the algorithm proposed in this paper more closely fits the ground truth trajectory than that of LOAM.The results verify the superiority of the proposed algorithm.

**Figure 14:**
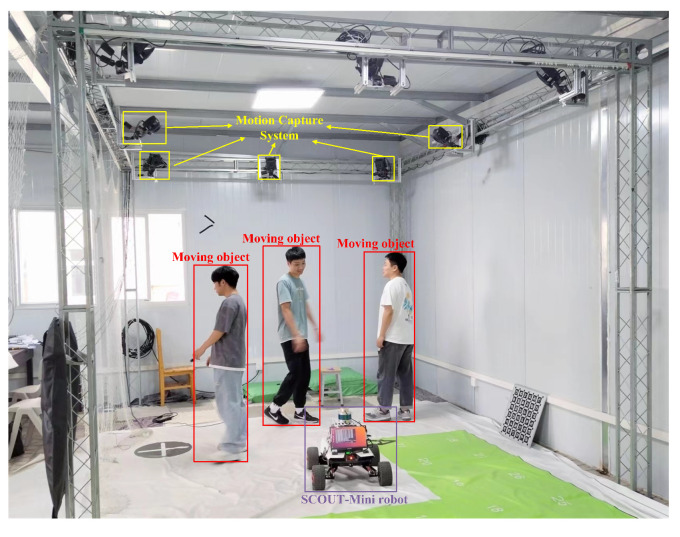
Realistic experimental test scenario.

**Figure 15:**
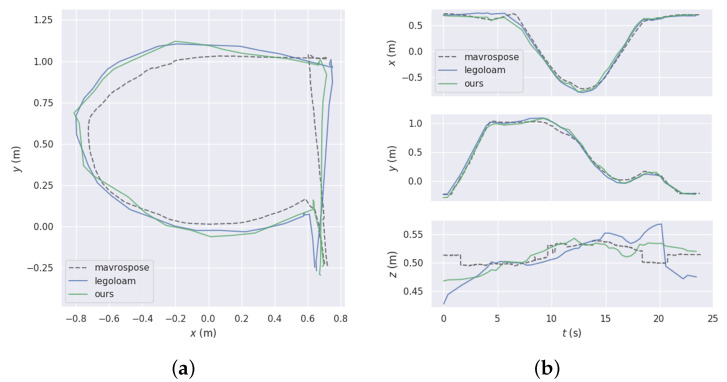
The results of the trajectory and localization error comparison in real scenarios. (**a**) Trajectory comparison results. (**b**) Positional error comparison results.

Table 4 shows the localization performance of the proposed algorithm compared to LeGO-LOAM in realistic scenarios, with reference values obtained using indoor motion capture data. As the table shows, the proposed algorithm has a more accurate localization performance compared to LeGO-LOAM. Similarly, the RMSE parameter in the RPE, indicating the local localization accuracy, is given in Table 5, verifying the stability of the algorithm proposed in this paper. The realistic experimental scenario is shown in Figure 14. A comparison of the trajectory and localization error in a small spatial area in a realistic scenario is shown in Figure 15, where “mavrospose” is the robot pose obtained by the motion capture system, which is used as the ground truth.

## 5. Conclusions

This paper investigates the interference of moving targets in the point cloud with the SLAM algorithm. Pre-processing operations are first performed, including ground point cloud removal, obstacle point cloud clustering, point cloud-to-image projection, and YOLO detection. The pre-processed point cloud and target detection information are used as input to the MoTI algorithm, and a probabilistic model is constructed by a method of information weighting and statistics during the two stages of rough and precise processing to enable accurate detection of moving targets. Through experiments with different datasets, the effectiveness of the MoTI algorithm for the detection of moving objects in point clouds is verified, and it is also demonstrated that the MoTI algorithm acting on the front-end of LOAM can improve the positioning accuracy and mapping effect of the system, which improve approximately 12.8% of the localization accuracy compared to LOAM. Multiple comparative experiments conducted on the KITTI dataset and the actual scenario dataset demonstrate the effectiveness of the proposed algorithm.

However, when the robot is relatively stationary with moving objects, it can be difficult to accurately identify them due to the insignificant change in depth difference. During the dataset processing, we observed that relatively stationary moving objects still retain some residual images in the point cloud map. In further planning, we will add further spatiotemporal correlation or loop closing to enhance the robustness of the system.

## Figures and Tables

**Table 1:** Accuracy of dynamic point cloud identification in adjacent three-frame point clouds.

Time Step	k	k + 1	k + 2
Dynamics number	819	839	805
Truth number	862	885	892
Identification rate/%	95.01	94.80	90.25

**Table 2:** ATE for full transformation pose relation (both translation and rotation errors are included) under different KITTI datasets.

Dataset Sequence	Algorithm	Mean	Median	min	rmse	std
00	LOAM	6.741	5.237	1.787	7.797	3.917
	LeGO-LOAM	5.591	4.675	2.287	6.385	3.184
	Proposed	6.200	4.357	2.284	6.382	3.112
06	LOAM	4.350	3.937	0.371	4.963	2.389
	LeGO-LOAM	7.649	7.761	2.525	8.029	2.442
	Proposed	4.334	3.975	0.402	4.890	2.265
10	LOAM	4.664	4.153	1.784	5.103	2.071
	LeGO-LOAM	5.400	4.306	2.318	6.619	3.827
	Proposed	4.577	3.933	2.370	4.137	1.280

**Table 3:** The RMSE of RPE w.r.t rotation angle in radians (rad) for delta = 1.0 (m) using consecutive pairs.

Dataset Sequence	LOAM	LeGO-LOAM	Proposed
00/rad	0.075	0.169	0.066
06/rad	0.054	0.144	0.045
10/rad	0.047	0.115	0.044

**Table 4:** ATE for full transformation pose relation in real scenarios.

Algorithm	Mean	Median	min	rmse	std
LeGO-LOAM	2.443	2.421	2.208	2.444	0.084
Proposed	2.327	2.328	2.191	2.348	0.063

**Table 5:** The RMSE of RPE w.r.t rotation angle in radians (rad) for delta = 0.10 (m) using consecutive pairs.

Algorithm	LeGO-LOAM	Proposed
RMSE/rad	0.456	0.426

## Data Availability

Not applicable.
